# Healthy urban environments for children and young people: A systematic review of intervention studies

**DOI:** 10.1016/j.healthplace.2015.09.004

**Published:** 2015-11

**Authors:** Suzanne Audrey, Harriet Batista-Ferrer

**Affiliations:** School of Social and Community Medicine, University of Bristol, 39 Whatley Road, Bristol BS8 2PS, United Kingdom

**Keywords:** Urban environment, Young people, Children, Systematic review, Health outcomes

## Abstract

This systematic review collates, and presents as a narrative synthesis, evidence from interventions which included changes to the urban environment and reported at least one health behaviour or outcome for children and young people. Following a comprehensive search of six databases, 33 primary studies relating to 27 urban environment interventions were included. The majority of interventions related to active travel. Others included park and playground renovations, road traffic safety, and multi-component community-based initiatives. Public health evidence for effectiveness of such interventions is often weak because study designs tend to be opportunistic, non-randomised, use subjective outcome measures, and do not incorporate follow-up of study participants. However, there is some evidence of potential health benefits to children and young people from urban environment interventions relating to road safety and active travel, with evidence of promise for a multi-component obesity prevention initiative. Future research requires more robust study designs incorporating objective outcome measures.

## Highlights

• Changes to the urban environment may improve the health of children and young people.
• Intervention studies comprise heterogeneous designs, participants and outcomes.
• There is some evidence to support road safety and active travel measures.
• A multi-component obesity prevention initiative showed evidence of promise.
• Future studies should incorporate robust study designs to assist policy decisions.

## 1 Introduction

Both globally and nationally, there has been increasing recognition of a need for action in building ‘healthy communities’ (Centers for Disease Control and Prevention, 2011, Edwards and Tsouros, 2006, National Institute for Health and Care Excellence, 2008, The Royal Town Planning Institute, 2009). Policy makers, urban planners and practitioners have a significant role in implementing and influencing policies to shape the urban environment in ways which enable people to live healthier lives. Historically, urban planning has focused on improving system efficiency or reducing environmental impacts (Handy et al., 2002). However, in more recent years there has been a focus on linking characteristics of the built environment with health behaviours and outcomes of the population.

There are increasing concerns that current ‘lifestyles’ in high income countries, particularly poor quality diet and sedentary behaviour, lead to chronic illnesses and health disparities which are socially and geographically patterned. Children can be particularly vulnerable as they have few opportunities to choose or change their environment. Systematic review evidence derived from cross-sectional or longitudinal studies have identified components of the built environment associated with physical inactivity (Sallis and Glanz, 2006), active travel (Pont et al., 2009), dietary intake (Sallis and Glanz, 2006), obesity (Dunton et al., 2009, Galvez et al., 2010), and mental health (Clark et al., 2007, Sinha and Rosenberg, 2013) in children and young people. Lack of sidewalks, distance to school or public open spaces, and density and availability of food sources are correlated with poorer physical health behaviours and outcomes (Dunton et al., 2009, Galvez et al., 2010, Pont et al., 2009, Sallis and Glanz, 2006). Worse mental health outcomes are associated with exposure to violence or crime in the neighbourhood (Clark et al., 2007, Sinha and Rosenberg, 2013). Although adaption of the built environment to overcome these factors may have the potential to improve health, robust intervention studies are required to provide evidence of a causal relationship and effectiveness.

A Cochrane review of built environment interventions for increasing physical activity in children and adults is yet to be published (Tully et al., 2013). However, in the protocol the authors argue that much of the previous evidence has been from cross-sectional studies which demonstrate inconsistent associations between features of the built environment and physical activity, do not demonstrate a causal relationship and do not control for confounders such as more active people choosing to live in neighbourhoods that support physical activity.

In a recent systematic review of the impact of interventions to promote physical activity in green space (Hunter et al., 2015) the authors argue, given the significant investment by local authorities in maintaining and improving urban green spaces, there is a need to identify if investments are effective in increasing the use of such spaces and whether there are public health benefits. Some evidence supported the use of built environment only interventions, but more promising evidence was found for physical activity programmes combined with changes to the built environment. However, the authors urged caution in interpreting the results because of the paucity of intervention-based research in this area. Furthermore, they highlighted the dearth of evidence in relation to children and adolescents.

The aim of the current systematic review is to examine evidence from intervention studies which involved changes to the urban environment and reported outcomes in relation to health related behaviours, and the physical or mental health outcomes, of children and young people.

## 2 Methods

The Preferred Reporting Items for Systematic Reviews and Meta-Analyses (PRISMA) guidelines were followed throughout the design, conduct, and reporting of this systematic review (Moher et al., 2009).

### 2.1 Search strategy

A comprehensive search strategy to identify primary studies reporting interventions to the urban environment and the health outcomes of children and young people was developed by an experienced systematic reviewer (H.B.-F.) for the Medline database. This was refined following discussion with the second reviewer (S.A.). A combination of the following Medical Subject Heading (MeSH) terms indexed within the database, and relevant text words from previous systematic reviews, comprised the initial search strategy: ‘Obesity’; ‘Weight gain’; ‘Weight loss’; ‘Diet’; ‘Dietary fats’; ‘Exercise’; ‘Physical activity’; ‘Mental disorders’; ‘Adjustment disorders’; ‘Anxiety disorders’; ‘Mood disorders’; ‘Neurotic disorders’; ‘Child’; Adolescent’; ‘Child, preschool’; ‘Infant’; ‘Urban health’; ‘City planning’; ‘Urban renewal’; ‘Environment design’; ‘Public facilities’; ‘Intervention studies’; ‘Evaluation studies’, and; ‘Program evaluation’ (Supplementary file 1). The indexing terms were modified to be applicable to other databases.

### 2.2 Data sources

The following biomedical, geographical, and transportation databases were searched from inception to 29 October 2014: Embase; Geobase; Medline; PsycINFO; Transportation Research Information Services, and; ISI Web of Science & ISI Proceedings. Searches were not restricted by date of publication. All abstracts were saved using reference manager Endnote X3.

### 2.3 Study selection

Intervention studies (randomised controlled trial, controlled trial, controlled before and after, before and after, interrupted time series) were eligible if they: included a change to the built environment; reported outcomes in relation to children and young people's physical or mental health and well-being, or health behaviours such as dietary intake or physical activity, or counts of active transport or park use; were undertaken in urban areas; were published in English, and; were undertaken in high-income countries using the World Bank classification (available from http://data.worldbank.org/about/country-classifications). Studies were excluded if they: focused on changes to the school or home environment rather than the wider public realm; were undertaken in rural areas or low- or middle-income countries, or; were not published in English. Conference abstracts, dissertations, letters, and books were not eligible for inclusion but were checked for relevant publications. Separate publications presenting results from the same intervention were reported together.

After duplicates were removed, all records were reviewed by one reviewer (H.B.-F.) to consider their relevance for inclusion. A random 10% sample of the records was independently assessed by a second reviewer (S.A.), with ‘very good’ inter-rater agreement (kappa=0.90) (Altman, 1991). Full text articles were retrieved and independently assessed for inclusion by two reviewers (H.B.-F. and S.A.). Disagreements were resolved by discussion. Reference lists and bibliographies from relevant primary studies, reviews, and intervention protocols were hand searched for additional primary studies not retrieved by the electronic search (H.B.-F.).

### 2.4 Data extraction

One reviewer (H.B.-F.) extracted and entered the following information for each study onto an excel spreadsheet: study characteristics (authors’ names, publication year, location, study period, objective(s), participants, and intervention setting), and; characteristics of study design (intervention, change to urban environment, sampling strategy, data collection methods, analysis, and main findings). These were double-checked by another reviewer (S.A.) to ensure accuracy.

### 2.5 Quality assessment

Quality assessment allows the methodical appraisal and evaluation of primary studies and is an established feature of systematic reviews of randomised controlled trials. Due to the anticipated nature of built environment interventions, which may preclude incorporation of randomisation and blinding within the study design, we did not exclude studies on the basis of quality. Quality assessment was carried out primarily to highlight the risk of bias and the resulting uncertainty of the results reported. Eligible primary studies were appraised by one reviewer (H.B.-F.) using a validated tool for non-randomised controlled trials (Sterne et al., 2014).

## 3 Results

A narrative synthesis approach (Popay et al., 2006) to reporting the results was taken because of the heterogeneity of outcomes, population groups and interventions. Further, there was a lack of suitable data to calculate standardised effect sizes (Higgins and Green, 2011). Primary studies were grouped according to the main focus of the intervention and reported narratively. Included primary studies are summarised in Table 1; the study designs and main findings are shown in Table 2, and; the assessment of bias is in Table 3. Table 4 offers a simplified overview of the key results for different types of intervention with their respective overall risk of bias.

### 3.1 Study selection

**Fig. 1:**
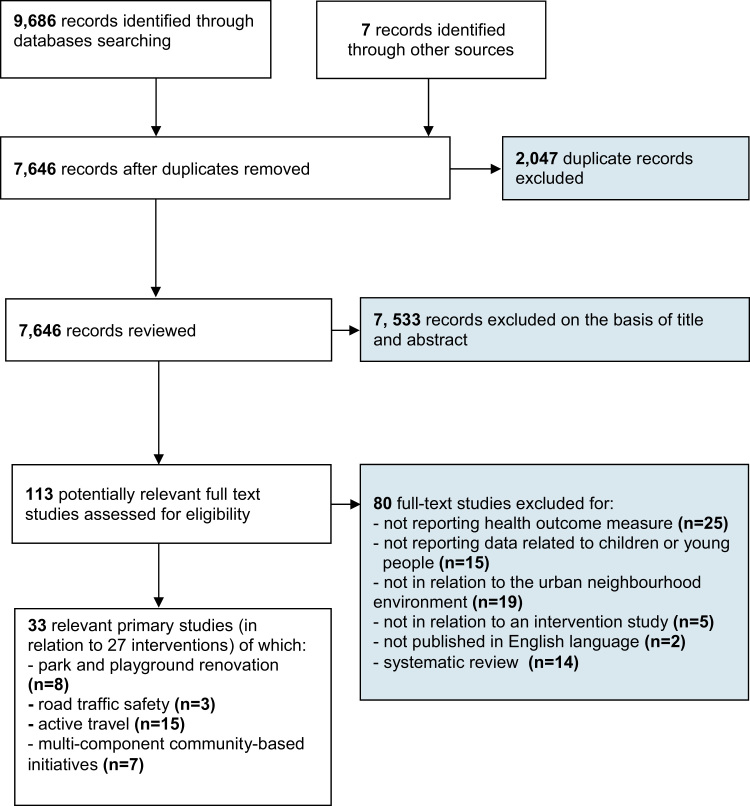
Flow diagram of study selection procedure.

Of 9686 records initially identified through the database searches, 7645 records were reviewed and 113 full text studies assessed for eligibility (Fig. 1). Of those full text studies excluded: 25 did not report a health behaviour or health outcome measure; 15 presented no data in relation to children or young people; 19 did not incorporate changes to the built environment; five were not intervention studies, and; two were not published in English. Fourteen systematic reviews were excluded following hand searches of reference and citation lists. A total of 33 relevant primary studies in relation to 27 separate interventions were included in this review.

### 3.2 Summary of included primary studies

Interventions were predominantly undertaken in the United States of America (USA) (*n*=17, 63.0%) with others conducted in Australia (4, 14.8%), New Zealand (2, 7.4%), Canada (2, 7.4%) and the United Kingdom (UK) (2, 7.4%). The majority of studies adopted a quasi-experimental pre-post evaluation design (21, 77.7%), of which eight (29.6%) included a comparison group. The remaining evaluation designs were observational (4, 14.8%), cluster randomised controlled (1, 3.7%), and A_1_–B_1_–A_2_ experimental design (i.e. establishing a baseline condition, introducing an experimental condition, and then reverting to the baseline condition) (1, 3.7%).

Eight interventions (29.6%) comprised modifications to parks and playgrounds, in which study participants included park users (6 studies), population-based samples (1 study), and school populations (1 study). The impact of changes to the built environment on road traffic safety measures was examined in three studies (11.1%) which all used population-based data sources. Of seven studies relating to four multi-component community-based health initiative interventions, six focussed on school populations and one considered the wider community. Finally, for the 12 interventions which aimed to promote active travel (44.4%, 15 studies), 13 studies selected participants from school populations and two focused on the wider community.

### 3.3 Parks and playgrounds

Of the eight studies in this category (Bohn-Goldbaum et al., 2013, Cohen et al., 2009, Cohen et al., 2012, Davidson et al., 1994, Quigg et al., 2012, Roemmich et al., 2014, Tester and Baker, 2009, Veitch et al., 2012), seven were in relation to park improvements which included: introduction of playgrounds (Bohn-Goldbaum et al., 2013, Cohen et al., 2009), gymnasiums (Cohen et al., 2009) or gym equipment (Cohen et al., 2009, Cohen et al., 2012); renovation of existing playgrounds (Cohen et al., 2009, Davidson et al., 1994, Quigg et al., 2012), and; park renovations (Bohn-Goldbaum et al., 2013, Cohen et al., 2009, Davidson et al., 1994, Tester and Baker, 2009, Veitch et al., 2012). In one study, the seating arrangement of the park environment was manipulated with the removal of seating during one experimental condition (Roemmich et al.,2014).

All studies were considered to be at an overall serious risk of bias. More specifically, individual domains measuring confounding (Bohn-Goldbaum et al., 2013, Cohen et al., 2009, Cohen et al., 2012, Roemmich et al., 2014, Tester and Baker, 2009, Veitch et al., 2012), selection of participants (Bohn-Goldbaum et al., 2013, Cohen et al., 2009, Cohen et al., 2012, Davidson et al., 1994, Quigg et al., 2012, Tester and Baker, 2009, Veitch et al., 2012), missing data (Bohn-Goldbaum et al., 2013, Cohen et al., 2009, Cohen et al., 2012, Roemmich et al., 2014, Tester and Baker, 2009, Veitch et al., 2012) and measurement outcomes (Bohn-Goldbaum et al., 2013, Cohen et al., 2009, Cohen et al., 2012, Roemmich et al., 2014, Tester and Baker, 2009, Veitch et al., 2012) were frequently assessed as at a serious risk of bias.

The sampling strategy for study sites was non-randomised for seven studies (Bohn-Goldbaum et al., 2013, Cohen et al., 2009, Cohen et al., 2012, Davidson et al., 1994, Quigg et al., 2012, Tester and Baker, 2009, Veitch et al., 2012), of which six incorporated comparison sites within the study design (Bohn-Goldbaum et al., 2013, Cohen et al., 2009, Davidson et al., 1994, Quigg et al., 2012, Tester and Baker, 2009, Veitch et al., 2012). One study did not report the sampling strategy (Roemmich et al., 2014). Objective measures captured included: Body Mass Index (BMI) (Quigg et al., 2012); Mean Total Daily Physical Activity using accelerometer data (Quigg et al., 2012), and; injuries and deaths using routinely collected hospital data (Davidson et al., 1994). The remaining studies (Bohn-Goldbaum et al., 2013, Cohen et al., 2009, Quigg et al., 2012, Roemmich et al., 2014, Tester and Baker, 2009, Veitch et al., 2012) used the System for Observing Play and Recreation in Communities (SOPARC) validated direct observational tool (McKenzie et al., 2006).

As part of a multi-component intervention, which included park and playground renovations in the USA, annual incidence rates of injuries in children aged 5–16 years decreased (relative risk: 0.74, 95% CI: 0.62–0.89) compared with the previous six years (Davidson et al., 1994).

Change to levels of park usage was reported in five studies (Bohn-Goldbaum et al., 2013, Cohen et al., 2009, Cohen et al., 2012, Tester and Baker, 2009, Veitch et al., 2012). In an Australian-based study, the number of children and teen park users increased between baseline and six-months post-intervention, although *p*-values were not provided (Veitch et al., 2012). In another Australian-based study, no detectable difference between the mean number of children using the parks was observed at the two year follow-up time point (*p*=0.42) (Bohn-Goldbaum et al., 2013). Three studies undertaken in the USA showed a decline in the number of children and adolescent park users post-intervention (*p*-values not provided) (Cohen et al., 2009, Cohen et al., 2012, Tester and Baker, 2009).

Interventions did not appear to increase children's or young people's level of physical activity. Following park renovations and upgrades in the Australia (Bohn-Goldbaum et al., 2013) and New Zealand (Quigg et al., 2012), no evidence was demonstrated for a difference in the proportion of children engaging in Moderate to Vigorous Physical Activity (MVPA) at the two year follow-up time point (*p*=0.73) (Bohn-Goldbaum et al., 2013) or for objectively measured Mean Total Daily Physical Activity after one year follow-up (*p*-value not provided) (Quigg et al., 2012). In a USA-based intervention, removal of seating arrangements in the park did not change the likelihood of children standing or engaging in MVPA (*p*=0.35) (Roemmich et al., 2014).

### 3.4 Road traffic safety measures

Three studies examined road traffic injuries (Dimaggio and Li, 2013, Grundy et al., 2009, Ragland et al., 2014) of which one UK-based study investigated the impact of 20 miles per hour (mph) traffic speed zones on road traffic casualties from a non-randomised observational study (Grundy et al., 2009). All three were classified at an overall moderate risk of bias with none of the individual domains being classified as at serious risk of bias.

The effect of the USA Safe Routes to School programmes (which included installation of sidewalks and traffic calming measures) on road traffic causalities and rate of collisions was also reported: data in relation to 30 of 124 potentially eligible intervention sites (Dimaggio and Li, 2013) and 93 of 313 Safe Routes to School programmes (Ragland et al., 2014) were obtained for these studies. For all three studies, outcome measures were population-based and comprised routinely collected data in relation to hospital admissions and police investigations for crashes and road traffic accidents.

Introduction of 20 mph traffic speed zones was associated with an annual average decline of 3.4% (95% CI: 3.1–3.7) in the incidence of all types of casualties amongst children aged 0–15 years and an annual average decline of 3.9% (95% CI: 3.6–4.3) reduction in pedestrian casualties aged 0–15 years (Grundy et al., 2009). Evidence for the impact of the Safe Routes to School programme was equivocal. A post-intervention decrease in the annual rate of school-aged pedestrian injury (44%, 95% CI: 17–65) during school-travel hours was reported in the New York programme (Dimaggio and Li, 2013). However, there was no strong evidence for a post-intervention reduction in collisions within 250 feet of a built environment change (incident rate ratio: 0.47, 95% CI: 0.20–1.12) in the Californian programme (Ragland et al., 2014).

### 3.5 Multi-component community-based initiatives

Seven USA-based studies in relation to four multi-component, community-based environmental change interventions were identified (Chomitz et al., 2012, Dunton et al., 2012, Economos et al., 2007, Economos et al., 2013, Folta et al., 2013, Hendricks et al., 2009, Maddock et al., 2006). Three primary studies were judged at an overall moderate risk of bias (Dunton et al., 2012, Economos et al., 2007, Economos et al., 2013), with the remainder being identified at serious risk of bias (Chomitz et al., 2012, Folta et al., 2013, Hendricks et al., 2009, Maddock et al., 2006). Individual domains identified as at serious risk of bias included measurement outcomes (Chomitz et al., 2012, Folta et al., 2013, Hendricks et al., 2009, Maddock et al., 2006), confounding (Hendricks et al., 2009, Maddock et al., 2006) and selection of participants (Hendricks et al., 2009, Maddock et al., 2006).

In the ‘Shape Up Somerville’ intervention, changes to the built environment included: traffic calming measures to and from the school environment; advocacy to paint crosswalks; installation of pedestrian crossing signs; opening and renovation of parks, and; provision of bike racks. Selection of the study site for intervention was non-randomised and included either two (Economos et al., 2007, Economos et al., 2013, Folta et al., 2013) or one (Chomitz et al., 2012) control site. Objectively measured BMI data was reported (Economos et al., 2007, Economos et al., 2013), in addition to parent-reported children's dietary and physical activity behaviours (Folta et al., 2013) and student self-reported levels of physical activity (Chomitz et al., 2012).

In comparison to the control communities, average change of the BMI *z*-score in the ‘Shape Up Sommerville’ intervention community was −0.10 (95% CI: −0.12 to −0.09) at the one year follow-up time point (Economos et al., 2007) and −0.06 (95%CI: −0.10 to −0.05) at the two year follow-up time point (Economos et al., 2013). Parent-reported data indicated children consumed less sugar-sweetened beverages (−2.0 ounces per day; 95% CI: −3.8 to −0.2) and were more likely to participate in organised sports and physical activities (0.20 sports or activities per year; 95% CI: 0.06–0.33) at the two year follow-up time point (Folta et al., 2013). Student self-report data at the four year follow-up time point suggested that high-school aged students were more likely to meet physical activity recommendations at follow-up after adjusting for demographic, health, and behavioural variables (OR: 2.36, 95% CI: 2.29–2.43) (Chomitz et al., 2012).

One study undertaken in California examined changes to physical activity following a move to a Smart Growth community, which was designed with features considered conducive to physical activity such as fewer barriers to connectivity, more parks and playgrounds, and traffic safety (Dunton et al., 2012). Using accelerometer data, there was no strong evidence that daily MVPA increased to a greater extent in the Smart Growth group (*p*=0.51) (Dunton et al., 2012).

Two of the community-based interventions used a before and after study design without a comparison group (Hendricks et al., 2009, Maddock et al., 2006). Changes to the built environment included improvements for pedestrian safety through the provision of walking paths and signs (Hendricks et al., 2009, Maddock et al., 2006). Using student self-report measures, increases in the proportion of students walking to school in Michigan were identified in four schools which had at least two years of data (Hendricks et al., 2009). However the Hawaii initiative showed, through self-report questionnaire data, that the proportion of students classified as overweight or at risk of being overweight increased and those consuming five portions of fruit and vegetables a day decreased at follow-up (Maddock et al., 2006).

### 3.6 Active travel

Fifteen primary studies (Boarnet et al., 2005a, Boarnet et al., 2005b, Buliung et al., 2011, Deehr and Shumann, 2009, Fitzhugh et al., 2010, Garrard and Crawford, 2010, Hinckson and Badland, 2011, Hinckson et al., 2011, Mammen et al., 2014a, Mammen et al., 2014b, McDonald et al., 2013, Morrison et al., 2004, Moudon and Stewart, 2012, Parker et al., 2013, Wen et al., 2008) in relation to 12 interventions were identified which aimed to increase the level of active travel amongst children and young people.

The majority of primary studies examined the impact of multi-component programmes which included: Safe Routes to School (Boarnet et al., 2005a, Boarnet et al., 2005b, Deehr and Shumann, 2009, McDonald et al., 2013, Moudon and Stewart, 2012); School Travel Plans (Buliung et al., 2011, Hinckson and Badland, 2011, Hinckson et al., 2011, Mammen et al., 2014a, Mammen et al., 2014b); Ride2School (Garrard and Crawford, 2010), and; Central Sydney Walk to School Research programme (Wen et al., 2008). Primary studies in relation to single component interventions included the addition of a bike lane in an urban area (Parker et al., 2013), retrofitting an urban greenway/trail (Fitzhugh et al., 2010), and introduction of a traffic calming scheme (Morrison et al., 2004).

Of the active travel interventions all studies were considered at serious risk of bias. The risk of bias due to confounding was considered serious in nine of the studies (Boarnet et al., 2005a, Boarnet et al., 2005b, Buliung et al., 2009, Deehr and Shumann, 2009, Fitzhugh et al., 2010, Garrard and Crawford, 2010, Morrison et al., 2004, Moudon and Stewart, 2012, Parker et al., 2013). The risk of bias due to selection of participants (Boarnet et al., 2005a, Boarnet et al., 2005b, Buliung et al., 2009, Deehr and Shumann, 2009, Fitzhugh et al., 2010, Garrard and Crawford, 2010, Hinckson and Badland, 2011, Hinckson et al., 2011, Mammen et al., 2014a, Mammen et al., 2014b, McDonald et al., 2013, Morrison et al., 2004, Moudon and Stewart, 2012, Parker et al., 2013) and measurement outcomes (Boarnet et al., 2005a, Boarnet et al., 2005b, Buliung et al., 2009, Deehr and Shumann, 2009, Fitzhugh et al., 2010, Garrard and Crawford, 2010, Hinckson and Badland, 2011, Hinckson et al., 2011, Mammen et al., 2014a, Mammen et al., 2014b, McDonald et al., 2013, Morrison et al., 2004, Parker et al., 2013) was frequently considered serious.

Prospective student surveys were most frequently used to capture information in relation to method of travel (Buliung et al., 2011, Garrard and Crawford, 2010, Hinckson and Badland, 2011, Hinckson et al., 2011, Mammen et al., 2014b, McDonald et al., 2013, Wen et al., 2008). Retrospective (Boarnet et al., 2005a, Buliung et al., 2011, Mammen et al., 2014a) and prospective parent surveys (Garrard and Crawford, 2010) were less frequently used. Four studies used non-validated observational methods to provide count data on method of travel (Boarnet et al., 2005b, Fitzhugh et al., 2010, Morrison et al., 2004, Parker et al., 2013). Two studies did not explicitly report the methods used to collect the primary outcome data (Deehr and Shumann, 2009, Moudon and Stewart, 2012).

#### 3.6.1 Safe Routes to School programme

There was some weak evidence to support the effectiveness of the Safe Routes to School programmes at increasing active travel. Across five USA states (Florida, Mississippi, Texas, Washington and Wisconsin), pre- and post-intervention data suggested that overall active travel increased from 12.9% to 17.6% (*p*<0.001). There was evidence that walking increased by 45% (9.8% pre-intervention, 14.2% post-intervention, *p*<0.001) and cycling increased by 24% (2.5% pre-intervention, 3.0% post-intervention, *p*=0.01) (Moudon and Stewart, 2012).

Similarly, through the Eugene programme (Oregon, USA), student reported data showed increased walking and bicycling to school after four years follow-up (number or *p*-values not reported) (McDonald et al., 2013). Sites which implemented a programme that combined education and two Safe Routes to School interventions were associated with a 20% increase in rates of walking (*p*≤0.05), but no associated affect with cycling (*p*-value not reported). However, there was no evidence for a difference in sites which implemented education and crosswalk/sidewalk interventions (*p*-values not reported) (McDonald et al., 2013).

In the Californian programme, observations of students walking to school suggested an increase in three of five sites which implemented sidewalk improvement projects and two of 10 sites which implemented traffic signals, but no differences were observed for crosswalk and signal or bicycle path improvement projects (Boarnet et al., 2005b). However, retrospective parent-reported data suggested there were more children who reduced their rates of walking or cycling (18.0%, 155/862) post-construction, than children who increased walking or cycling (10.6%, 91/862) (Boarnet et al., 2005a). In Seattle, the authors reported a 24% increase in number of students who walked to school (data collection method and *p*-values not reported) where speed limits were enforced (Deehr and Shumann, 2009).

#### 3.6.2 School Travel Plan programme

Evidence for the effectiveness of the School Travel Plan programme at increasing active travel was inconsistent. In a Canadian programme, child self-report data showed a small increase in active transport (pre-intervention: 43.8%, post-intervention: 45.9%, *p*-value not reported) (Buliung et al., 2011). In another Canadian programme, there was no evidence for a difference in active travel using child self-report data (*n* and p-values not provided) (Mammen et al., 2014a) but retrospectively collected parental data suggested reductions in driving of 16.7% in the morning (*n*=1,118) and 17.1% in the afternoon (*n*=1,211) (Mammen et al., 2014b). Infrastructure improvements and safety education were perceived by parents to be the most effective strategies implemented (Mammen et al., 2014a). In the New Zealand programme, student-reported data suggested an increase in active travel at the second (5.9%, standard deviation (SD)±6.8%) (Hinckson and Badland, 2011) and third (OR: 2.65, 95% CI:1.75–4.02) year time points (Hinckson et al., 2011).

#### 3.6.3 Ride 2 School programme

The authors reported a small increase in the proportion of active trips to and from school using parent-reported data (47.9% pre-intervention, 49.6% post-intervention, *p*-values not reported) but student-reported data suggested a decrease (51.1% pre-intervention, 48.7% post-intervention, *p*-values not reported) (Garrard and Crawford, 2010).

#### 3.6.4 Central Sydney Walk to School Research programme

Parent-reported data from a randomised controlled trial showed a net increase of 9.8% (*p*=0.05) of students increasing active travel in the intervention group at the one year-follow up time point. However, there was no evidence for differences in active travel by student-reported data. Distance from home to school and non-car use at baseline were predictors of non-car use at the one year follow-up time point (both *p*<0.001) (Wen et al., 2008).

#### 3.6.5 Single component interventions

Following the introduction of a cycle lane in an urban area in the USA, the mean number of youths observed cycling each day doubled (baseline 2.2, SD 3.1; 1-year follow-up 5.2, SD 7.4) (Parker et al., 2013). However, no differences were found in median counts of active travel in experimental schools between baseline (8.5) and the two year follow-up time point (9.0) (*p*-values not provided) after the retrofitting of urban greenway/trail in Knoxville, USA (Fitzhugh et al., 2010). In a UK traffic calming scheme (comprising speed cushions, zebra crossings and parking bays), there was strong evidence that the observed pedestrian count across the road in which the intervention was implemented in all three sites (Morrison et al., 2004).

## 4 Discussion

### 4.1 Main findings

This study systematically reviewed the available literature on interventions which incorporated changes to the built environment and reported health behaviours or outcomes for children and young people. There was some evidence of promise for interventions to reduce road traffic injuries and interventions to increase young people's active travel to school, and in relation to a multi-component obesity prevention health initiative. There was limited evidence that interventions to parks and playgrounds increased usage.

A diverse range of study designs, outcome measures and study settings were used in the primary studies. Evidence for effectiveness of such interventions is at present weak and compounded by the study designs which may be opportunistic, frequently incorporate non-randomised allocation of study sites, use subjective outcome measures, and do not incorporate follow-up of study participants. The uncertainty in the evidence currently limits our understanding of which changes to the built environment can improve health behaviours and outcomes of children and young people. This in turn creates challenges in developing specific recommendations for policy makers and the public sector to make decisions in relation to the implementation of urban development projects.

### 4.2 Comparison to the literature

#### 4.2.1 Road traffic safety

There was evidence from two studies that interventions may reduce road traffic injuries in children and young people and create safer communities. These studies had access to relatively robust routinely collected, population-based data for longer time periods than studies relating to other interventions. Similarly, in relation to the general population evidence from two systematic reviews and meta-analyses suggests that area-wide urban traffic calming schemes, such as speed limits and one way systems, on average reduce the number of injuries by about 15% (Elvik, 2001) and can reduce road crash related deaths (pooled rate ratio: 0.63, 95% CI: 0.14–2.59) (Bunn et al., 2003).

#### 4.2.2 Active travel

Accessible pavements and street connectivity are considered important to facilitate active travel. A recent systematic review examining interventions which promoted active travel to school (including educational programmes without any changes to the built environment), found only three of the 14 interventions had a large, or very large, effect size on rates of active travel (Chillón et al., 2011). In the current systematic review, 13 of the 15 primary studies identified suggested an increase in active travel (Boarnet et al., 2005a, Boarnet et al., 2005b, Buliung et al., 2011, Deehr and Shumann, 2009, Garrard and Crawford, 2010, Hinckson and Badland, 2011, Hinckson et al., 2011, Mammen et al., 2014a, McDonald et al., 2013, Morrison et al., 2004, Moudon and Stewart, 2012, Parker et al., 2013, Wen et al., 2008) but the findings need to be treated with caution: some studies did not provide evidence for size of effect (Deehr and Shumann, 2009, Fitzhugh et al., 2010, Garrard and Crawford, 2010, McDonald et al., 2013, Moudon and Stewart, 2012), reported very small effects (Buliung et al., 2011, Garrard and Crawford, 2010, Hinckson et al., 2011), or used retrospective parental report methods (Boarnet et al., 2005a, Hinckson et al., 2011).

Many of the changes to the built environment in the primary studies of the current review were implemented as part of a multi-component programme. Therefore, attributing behavioural outcomes to specific changes to the built environment is difficult to untangle. In addition, few authors attempted to address this in the analysis. None of the studies used objective measures of active travel, such as accelerometer data. This is a significant weakness given that there were two studies in which parents and children, participating in the same study, self-reported opposing results (Garrard and Crawford, 2010, Wen et al., 2008).

#### 4.2.3 Park and playground interventions and physical activity

It is argued that the provision of clean, safe, and accessible public open spaces can offer children and young people opportunities for physical activity and social interaction (National Institute for Health and Care Excellence, 2008). However, the limited findings from this review, focussing on the public realm, suggest minimal effects of playground and park interventions in creating positive changes to usage or physical activity levels in children and young people.

The effect of physical changes to the playground environment has been more frequently researched in relation to the school setting. Systematic review evidence has shown that manipulation of pre-school playground environment with markings or equipment (Temple and Robinson, 2014) and school playground markings plus physical structures (Escalante et al., 2014) increased physical activity levels in children and young people. It may be that children are exposed to the intervention more often in the school environment compared to public parks which they may visit infrequently. Additionally in the school environment, researchers may find it easier to implement more robust study designs which incorporate follow-up of participants, as children are likely to attend the school for several years.

In the pre-school environment, a systematic review and meta-analysis showed that physical activity interventions which included environmental changes had a larger effect on increasing pre-school children's physical activity than interventions solely focused on physical activity (Gordon et al., 2013). Similarly, a systematic review examining the impact of interventions in children and adults to promote physical activity in urban green space, suggested combining changes to the built environment with physical activity programmes could be more effective than changes to the built environment alone (Hunter et al., 2015). This suggests future robust studies are required to evaluate the impact of combining structural improvements to public parks and playgrounds with behavioural change interventions.

## 5 Strengths and limitations

We followed a systematic and comprehensive process including: a search strategy applied to multiple databases in different research fields to uncover all relevant studies; a diverse range of eligible health outcome measures, and; no restrictions on the basis of publication date.

Nevertheless, there are a number of limitations. Publication bias may be present if interventions to the built environment that did not show positive results are less likely to have been submitted or accepted for publication. To allow comparability between studies, interventions undertaken in low- or middle-income countries were not eligible and the applicability of the present study findings in these settings is unknown. English language publication bias may also be present as studies not published in English were excluded.

The majority of primary studies were considered to be at serious risk of bias. Many of the studies used quasi-experimental designs, and the incorporation of statistical analyses that controlled for confounding variables was infrequent. Where possible, future interventions should incorporate experimental studies with a randomised controlled design at the level of the study site (e.g. park, school or city). Only two studies in this review used objective measures of physical activity. Future intervention studies should incorporate objectively measured outcomes: for example, a study using Geographical Information System (GIS) and accelerometers (Coombes et al., 2014) was able to show that children attending schools with a more supportive local environment were more likely to maintain active travel behaviours than those with less supportive environments. In addition, given the complexity of public health interventions, researchers should undertake process evaluation alongside implementation (Moore et al., 2015) to gain greater understanding about which parts of multi-component interventions are most effective in improving the health outcomes and behaviours of children and young people.

The built environment is thought to be an important contributory factor to the persistence of health inequalities in the UK (Glasgow Centre for Population Health, 2013). This suggests addressing the ‘upstream’ social determinants of health by making changes to the built environment has the potential to reduce population-wide health inequalities (Marmot et al., 2010, Marmot et al., 2008). Addressing barriers and facilitators operating at policy and neighbourhood levels, is likely to be a more effective and far reaching strategy for public health improvement than short-term, behavioural interventions targeted at the inter- or intra-personal level alone (McLeroy et al., 1988). Despite this, few studies examined or controlled for differences by socioeconomic status, ethnicity or gender. Therefore, the potential impact of built environment interventions on reducing inequalities remains unknown. Future studies need to be adequately powered to allow exploration of different effects amongst different population groups.

No primary studies reporting mental health or well-being outcomes were captured in this review. This is important because associations between poor mental health outcomes and exposure to community violence or crime have been shown (Clark et al., 2007, Sinha and Rosenberg, 2013). In addition, the UK governmental strategy on the built environment and health recognises that population improvements to mental health and well-being need to be evaluated, in addition to physical health outcomes such as obesity and unintentional injury (The Scottish Government, 2008). Further research is required to examine whether changes to the built environment can have positive effects on children's mental health and well-being outcomes.

## 6 Challenges and recommendations

Ogilvie et al. identified a number of challenges in evaluating the provision of new walking and cycling infrastructure, including the selection of variables to be measured for both exposure and outcomes, and suggest this relates in part to the different perspectives of public health and transport research (Ogilvie et al., 2012). This challenge was also evident in the studies included in the current review. For example, undertaking a head count in a park or playground at two time points may provide evidence of increased use, but is not sufficient to conclude an increase in children's physical activity or a reduction in BMI. However, the difficulty in attributing health outcomes should not be a reason to suggest local authorities should refrain from improving parks. Increased park use may be beneficial for reasons other than physical activity and a wider perspective may be necessary when considering effectiveness and cost effectiveness. Wildlife, education, safety or crime reduction may all be relevant or an urban park may, for example, assist storm water management (Hartig et al., 2014). Nevertheless, if the purpose is to improve health, then a robustly measured health outcome is required.

Hunter et al. (2015) list a number of methodological considerations to inform the design, implementation and evaluation of physical activity interventions in urban green spaces which are relevant to the wide range of studies identified in this review: reporting and justifying sample size and accounting for clustering where appropriate; using multiple data sources (observations, surveys, interviews, and objective measures such as accelerometer and GPS data); collecting baseline and long-term follow-up data, and; identifying an adequate (albeit not perfect) control condition. We would also highlight the need to consider and minimise the risk of bias. The tool used in this review (Sterne et al., 2014) is stringent and requires that a judgement of serious risk of bias within any domain should be applied to the study as a whole, irrespective of which domain is being assessed (Table 3). The tool has been developed for non-randomised studies of interventions and, while built environment interventions pose particular challenges, it is important to be aware of and minimise bias due to confounding, participant selection, measurement of interventions, departure from intended interventions, missing data, measurement of outcomes, and selection of reported results.

Elsewhere, it has been argued that postponing action until a strong evidence base has been developed has the potential to cause more harm than good (Frank et al., 2012). Policy makers will continue to make decisions about the neighbourhood environments in which children and young people live, while public health researchers and transport or urban planners differ in their opinions about what constitutes evidence of effectiveness. It is important that inter-disciplinary teams share expertise across transport, planning, public health and other relevant disciplines. In the UK, the decision to locate public health within local authorities offers the potential for greater collaboration in building an evidence base that is sufficiently ‘robust’ for a public health audience while not inhibiting policy makers and planners.

We suggest that public health researchers, policy makers and the public sector should embrace opportunities to work collaboratively to implement and evaluate built environment interventions which address some of the methodological weaknesses identified in this review.

## 7 Conclusion

The interventions captured in this review related to active travel, park renovations, road traffic safety, and multi-component community health initiatives. Although the majority of studies had a serious risk of bias, there was some evidence of effectiveness of in relation road safety measures and active travel. Future research studies should involve collaborations between researchers, policy makers and planners, and consider using randomised controlled study designs which incorporate objective outcome measures. A joint agenda to generate and act upon the best available evidence will aid decision-making for those who must choose between, or prioritise, different options to improve the health and well-being of children and young people who live in urban environments.

## Conflicts of interest

The authors have no conflicts of interest to report.

## Figures and Tables

**Table 1:** Summary of included primary studies.

**Author**	**Publication year**		**Location**	**Study period**	**Study objective(s)**			**Study design**		**Health related outcome(s)**	**Participants**	**Intervention setting**
Cohen et al. (2012)	2009		Southern California, USA	December 2003–March 2008	To assess the impact of park improvements on park use and physical activity			Quasi-experimental pre-post evaluation design with a comparison group		Count of park users	Children and adolescent park users	5 parks
Cohen et al.	2012		Los Angeles, USA	Winter 2008 –Spring 2010	To evaluate the impact of the Fitness Zone outdoor exercise equipment on physical activity in parks			Quasi-experimental pre-post evaluation design with a comparison group		Count of park users	Children and adolescent park users	22 parks
Davidson et al.	1991		New York, USA	1983–1991	To evaluate the effectiveness of a community coalition to prevent severe injuries to children			Observational study of a quasi-experimental pre-post evaluation design with a comparison group		Injury incidence rates in school-aged children and infants	Newborn to 4-year olds and children aged 5–16 years with fatal and non fatal injuries	5 parks and playgrounds
Quigg et al.	2012		Dunedin, New Zealand	October 2007–December 2008	To assess whether an upgrade of playgrounds in a community was associated with changes in the physical activity of local children			Quasi-experimental pre-post evaluation design with a comparison group		Mean Total Daily Physical Activity	*Baseline*: 184 children aged 5 to 10 years attending 8 schools in the community	2 playgrounds
*1 year follow-up*: 156 (86%)
Roemmich et al.	2014		Grand Forks, North Dakota, USA	July 2012–August 2013	To test whether a micro-environment park intervention would increase the physical activity and length of stay of park users			Experimental design (A_1_–B_1_–A_2_)		Child physical activity intensity	Children park users	1 park
Tester et al.	2009		San Franciso, USA	May 2006–June 2007	To study the impact of a playfield renovation in two urban parks in low-income neighbourhoods			Quasi-experimental pre-post evaluation design with a comparison group		Count of park users	Children and teen park users	2 parks
Veitch et al.	2012		Victoria, Australia	August 2009–August 2010	To examine whether improvements to a park increased its use and park-based physical activity of users			Quasi-experimental pre-post evaluation design with a comparison group		Count of park users	Children and adolescent park users	1 park
**Road traffic safety measures**												
Dimaggio et al.	2013		New York City, USA	2001–2010	To analyse motor vehicle crash data to assess the effectiveness of Safe Routes To School interventions in reducing school-aged pedestrian injury			Observational study of quasi-experimental pre-post evaluation design with a comparison group (30 intervention sites and 1347 comparison groups)		Rates per 10,000 population for pedestrian injuries, rate differences and proportion changes	4760 school-aged pedestrian crashes	Population-based
Bohn-Goldbaum et al.	2013		Sydney, Australia	May 2007 –May 2009	To determine how a playground renovation impacts usage and physical activity of children			Quasi-experimental pre–post evaluation design with a comparison group		Daily mean number of children visiting playgrounds and the proportion of children engaging in MVPA	Children park users aged 2–12 years	1 park
**Parks and playgrounds**												
Grundy et al.	2009		London, UK	1986–2006	To quantify the effect of the introduction of 20 mph (32 km an hour) traffic speed zones on road collisions, injuries, and fatalities			Observational study		Annual average casualties and collisions on roads	119,029 road segments with at least one casualty	Population-based
Ragland et al.	2014		California, USA	1998–2009	To assess the long-term impact of programme funded engineering modifications on walking/bicycling levels and safety			Observational study of quasi-experimental pre–post evaluation design with a comparison group		Change in rate of collisions within 250 feet of the counter measure	Collision data involving pedestrians/bicyclists aged 5–18 (number not reported)	Population-based data in relation to collisions which were within 250 feet of improvements implemented by Safe Routes To School programme or quarter-mile school buffer zones
**Multi-component, community-based initiatives**												
Dunton et al.	2012		California, USA	May 2009–July 2010		To determine whether children change the type of contexts where they engage in physical activity after a recent move to a Smart Growth community		Quasi-experimental pre-post evaluation design with a control group		Objectively measured physical activity (Actigraph)	*Baseline:* 120 ethnically diverse children aged 9–13 years	Smart Growth communities
*6-month follow-up:* 102 children
Economos et al.	2007		Three culturally diverse urban cities, Massachusetts	September 2002–August 2005 (Economos et al., 2007, 2013; Folta et al., 2013)		To test whether a community-based environmental change intervention could prevent undesirable weight gain in children at 1 and 2 year follow-up (Economos et al., 2007, 2013)		Quasi-experimental: Non-randomised controlled trial (one intervention site and two control sites) (Economos et al., 2007, 2013; Folta et al., 2013)		Objectively measured BMI (Economos et al., 2007, 2013)	*Baseline:* 5940 potentially eligible students enroled in grades aged 6–8 years, 1696 (29%) consented to participate	30 schools- and the surrounding community- and home-environments
Economos et al.	2013	
Folta et al.	2013		Parent reported fruit and vegetable and sugar-sweetened beverage consumption; number of organised sports and physical activities per year; walking to and from school (Folta et al., 2013)
*1 year follow-up*: Data available for 1178 (69.5%) children
2003–2008 (Chomitz et al., 2012)	
Quasi-experimental: Non-randomised controlled trial (one intervention site and one control site) (Chomitz et al., 2012)	
Chomitz et al.	2012	
*2 year follow-up:* 1028 (60.6%) children (Economos et al., 2013)
To describe the behavioural changes in children resulting from Shape Up Somerville (Folta et al., 2013)	
Student self-reported achievement of either moderate or vigorous physical activity guidelines (Chomitz V, et al. 2012)	*2 year follow-up:* 454 parents of children (Folta et al., 2013)
To evaluate the Active Living by Design project implemented in Somerville (Chomitz et al., 2012)		*Baseline*: 1098 (90%) middle- and 1382 (81%) high-school students
*4 year follow-up*: 926 (88%) middle- and 1125 (79%) high-school students
Hendricks et al.	2009		Michigan, USA	2003–2006		To create a walkable community where all residents could engage in physical activity in a safe environment		Quasi-experimental: Multilevel community intervention, without comparison group		Proportion of children who walk to school at schools involved in the Safe Routes to School programme	Elementary school children (N not reported)	11 elementary schools
Maddock et al.	2006		Hawaii, USA	2000–2004		To create and implement a population-based intervention for physical activity and nutrition		Quasi-experimental: Multilevel community intervention, without comparison group		Student self-reported overweight or at-risk for overweight; consumption of fruit vegetables; engagement in moderate physical activity for a least 30 minutes per day	Middle and high school students (N not reported)	All middle- and high-schools (N not provided)
**Active travel**												
Boarnet et al. (2005a)	2005		California, USA	Spring 2002–Autumn 2003		To assess whether Safe Routes 2 School Programmes that focus on built environment changes can increase active travel to school (Boarnet et al., 2005a)			Quasi-experimental design with retrospective post-test assessment without a comparison group (Boarnet et al., 2005a)	Number of children who walked or bicycled to school after Safe Routes to School programme	*1 year follow-up:* 1244 of 1778 parents of children aged 8–11 years (response rate 39%)	10 elementary schools that implemented traffic improvement projects as part of the California Safe Routes to Schools programme
Boarnet et al. (2005b)	2005	
To evaluate California’s pioneering Safe Routes to Schools construction programme (Boarnet et al., 2005b)		
Quasi-experimental pre–post evaluation design without a comparison group (Boarnet et al., 2005b)
Buliung et al.	2011		Alberta, Nova Scotia, Ontario, British Columbia provinces, Canada	March 2008–April 2009		To conduct a pilot School Travel Planning intervention			Quasi-experimental pre–post evaluation design without a comparison group	Rates of active school transport participation	*Baseline:* Convenience sample of all students (N not reported)	12 schools which all received the intervention
*1-year follow-up:* Number of students not reported; Of 5012 students in participating schools, 1489 (30%) parents returned questionnaires
Deehr et al.	2009		5 neighbourhoods in Seattle, USA	2005–2007		To campaign for and implement strategies for active living within populations with a diversity of age, socioeconomic, and health characteristics			Quasi-experimental: Multilevel community intervention, without comparison group	Percentage of students who walked to school	Elementary aged students (age or N not reported)	1 elementary school that implemented the intervention (nested within larger, community-wide walkability programme)
Fitzhugh et al.	2010		Knoxville, USA	March 2005–March 2007		To examine the impact of neighbourhood connectivity on physical activity			Quasi-experimental pre–post evaluation design with comparison group	Observation counts of active transport to school	School-aged children in participating schools	One intervention neighbourhood
Garrard et al.	2010		Victoria, Australia	2007		To describe key findings from the evaluation of the Ride2School programme			Quasi-experimental pre-post evaluation design without a comparison group	Changes in rates of active travel to school	*Baseline*: 479 students and 409 parents	13 primary schools
*Approximately 8 months follow-up*: 403 students and 358 parents
Overall response rate: 29%
Hinckson and Badland (2011)	2011		Auckland region, New Zealand	2004–2008		To determine the effectiveness of the School Travel Plan programme in changing school travel modes in children (Hinckson and Badland, 2011)			Quasi-experimental pre–post evaluation design without a comparison group	Changes in rates of active travel to school	13,259 students aged 5–10 years (Hinckson and Badland, 2011)	33 elementary schools that implemented the School Travel Plan programme (Hinckson and Badland, 2011)
Hinckson et al. (2011)
56 elementary schools that implemented the School Travel Plan programme (Hinckson et al., 2011)
To describe trends in active commuting to school in children following implementation of the School Travel Plan programme (Hinckson et al., 2011)		
*Baseline:* 16,686
*1-year follow-up:* 17,494
*2-year follow-up:* 15,635
*3-year follow-up:* 7281 students aged 5–10 years (Hinckson et al., 2011)
Mammen et al. (2014a)	2014		Canada	January 2010–March 2012		To evaluate the Canadian School Travel Planning intervention by examining changes in school travel mode and predictors of mode change (Mammen et al., 2014a)			Quasi-experimental pre-post evaluation design without a comparison group (Mammen et al., 2014a)	Proportion of students who changed to an Active Travel mode (Mammen et al. 2014a)	*Baseline and 1 year follow-up:* Children aged 6 to 14 years old (number not provided) (Mammen et al., 2014a)	53 schools that participated in the School Travel Plan programme
Mammen et al. (2014b)
*1 year follow-up:* 7827 of 24,893 (31.4%) families attending schools that implemented School Travel Plan programme (Mammen et al., 2014b)
Proportion of parents who reported driving less (Mammen et al., 2014b)
To evaluate the Canadian School Travel Planning intervention by examining child-, family-, and school-level characteristics (Mammen et al., 2014b)		
Quasi-experimental retrospective post-test assessment without a comparison group (Mammen et al., 2014b)
McDonald et al.	2013		Eugene, Oregon, USA	2007–2011		To evaluate the impacts of a Safe Routes to School programme on walking and biking			Quasi-experimental pre–post evaluation design with a comparison group	Proportion of students walking and biking for school travel	Students of participating schools grades 1–8 at baseline (*n*=1582), 1 year (2303), 2-year (1032), 3 year (1121) 4-year (1372) follow-up	9 schools
Morrison et al.	2004		Glasgow, Scotland	June 2000–June 2001		To assess the secondary health and health related impacts on a local population after the introduction of a traffic calming scheme			Quasi-experimental pre–post evaluation design without a comparison group	Changes in pedestrian counts	Pedestrians walking on intervention road	Main road of intervention community
Moudon et al.	2012		Florida, Mississippi, Texas, Washington, and Wisconsin, USA	2005–April 2011		To assess the Safe Routes to School programme in five states			Quasi-experimental pre–post evaluation design without a comparison group	Changes in rates of active travel to school	53 schools that implemented the Safe Routes to School programme and provided data	Schools with pre- and post- Active Travel to School project data
*Baseline:* 73,344 students (N at follow-up not reported)
Parker et al.	2013		New Orleans, USA	September 2009–September 2010		To examine the impact of building new bike lanes and determine whether more people were cycling on the street and with the flow of traffic after bike lanes were built			Quasi-experimental pre–post evaluation design without comparison group	Number of people observed cycling	Youth cyclists at baseline and follow-up	Street with new bike lane
Wen et al.	2008		Inner west Sydney, Australia	March 2005–October 2006		To evaluate the effectiveness of a programme to increase walking to and from school			Cluster randomised controlled trial	Percentage of students who walked to and from school	*Baseline*: 1966 of 2258 students (87%) and 1606 parents (71%)	12 primary public schools
*1-year follow-up*: 1975 of 2232 students (88%) and 1362 parents (61%)

**Table 2:** Characteristics of study designs in primary studies.

**Author**	**Intervention**			**Change to built environment**	**Sampling strategy**	**Data collection methods**	**Analysis**	**Main findings**

**Parks and playgrounds**								
Bohn-Goldbaum et al.	Park and playground renovations			*Park renovation*: Upgrading paths, adding new greenery, lighting and facilities (e.g. park furniture)	*Study sites*: Non-randomised, park eligible for renovation and control park selected for similar characteristics	System for Observing Play and Recreation in Communities (SOPARC) for 2-week periods at baseline and after 2 years follow-up	Generalised linear model	*Park usage*: No detectable difference between the mean number of children at 2-year follow-up time point (interaction between park and time *p*=0.42); no differences by gender (*p*=0.97)
*Children and adolescents*: All observed
*Engagement in MVPA*: No detectable difference in children between parks at 2-year follow-up time point (interaction between park and time *p*=0.73); in the intervention park there was a decline in girls engaging in MVPA (*p*=0.04)
*Playground renovation*: Three unfenced playgrounds dispersed throughout the park
Cohen et al. (2009)	Park improvements			*Parks 1–3*: Completely new gymnasiums constructed	*Study sites*: Non-randomised, 5 parks eligible for upgrading funding and 5 matched parks not eligible	System for Observing Play and Recreation in Communities (SOPARC) for 7 days at baseline and between 3 and 14 months post-intervention	Propensity score analysis	*Park usage*: Number of children (6449–4717) and adolescent (3459–3387) park users declined post-intervention (*p*-values not provided)
*Park 4*: Gymnasium refurbished and underwent some field improvements in watering and landscaping
*Park 5*: Improvements to picnic areas, upgrades to a walking path, and enhancements to a playground area

*Children and adolescents*: All observed

Cohen et al. (2012)	Provision of outdoor exercise equipment			Introduction of outdoor exercise equipment	*Study sites:* Non-randomised, 12 parks selected for upgrade and selection of 12 similar parks that did not receive the equipment	System for Observing Play and Recreation in Communities (SOPARC) for 4-day periods at baseline, 12 months post-intervention and the following Spring	Descriptive analysis	*Park usage*: Number of children and adolescent park users at baseline (1381 and 787, respectively) declined post-intervention at 12 months (330 and 157) and second follow-up (244 and 121) (*p*-values not provided)
Davidson et al.	Safe Kids/Healthy Neighbourhoods injury prevention programme			Renovation of parks and playgrounds	*Study sites:* Non-randomised, one health district in New York City selected for upgrade of all parks and playgrounds and selection of contiguous area with no upgrade	Hospital surveillance data of injuries and death during the intervention period (1989–1991) and the pre intervention period (1983–1988)	Poisson regression model	During the intervention period, adjusted annual incidence rates of injuries in children aged 5–16 years decreased (relative risk:0.74, 95% CI: 0.62–0.89) compared with preintervention period. In the younger, nontargeted age group, no significant reduction in incidence occurred (relative risk:1.06, 95% CI:0.83–1.35)
Quigg et al.	Playground upgrades			*Playground 1*: 10 new components (play equipment, seating, additional safety surfacing, and waste facilities) were installed and 2 existing components were removed	*Study sites*: Non-randomised, community selected for upgrade and selection of similar control site	Accelerometer (Actigraph) for 6 days at baseline and after 1-year follow-up	Linear mixed model	*Physical activity*: No evidence for differences in mean Total Daily Physical Activity between intervention and control community (*p*-value not provided) at 1-year follow-up time point
There was evidence of an interaction between BMI and those exposed to the upgraded playgrounds; those with higher BMI were more likely to reduce physical activity in the intervention community (*p*=0.006)
*Schools*: 4 of 6 eligible schools in intervention community agreed to participate; all 4 eligible schools in control community agreed to participate
Objectively measured BMI at baseline and 1-year follow-up
*Children*: All children meeting eligibility criteria with parental consent
*Playground 2*: 2 new play equipment pieces were installed, and a small modification made to existing equipment
Roemmich et al.	Manipulation of location of seating around a park playground			Manipulation of location of seating around a park playground (A: Usual seating arrangement; B: Seating removed)	*Study site*: Assignment of park not described	System for Observing Play and Recreation in Communities (SOPARC) for 7 days for each of the study conditions at two time points 1-year apart	ANOVA and hierarchical linear model	*Physical activity*: Odds of children standing or being in MVPA rather than sitting were not associated with intervention condition B (seating removed) (*p*≥0.35)
Tester et al.	Renovation of parks			In both parks, artificial turf replaced uneven dirt fields, and new fencing, landscaping, lighting, and picnic benches were added. In Park A, permanent soccer goals were installed, and in Park B, a walkway around the field was restored	*Study site:* Non-randomised, 2 intervention parks were selected on: condition, typical use, ability to increase field capacity with artificial turf, community value of the parks and existing programming. Control park matched for similar characteristics	System for Observing Play and Recreation in Communities (SOPARC) for 6 days for each of the study condition at two time points 1 year apart	Descriptive analysis and independent *t*-tests	*Park usage:* Number of children and teen park users at baseline (28 and 110, respectively) increased post-intervention at 12 months (460 and 197, respectively) (*p*-values not provided)
Veitch et al.	Refurbishment of park			Establishment of a fenced leash-free area for dogs; an all-abilities playground; a 365-m walking track; a barbecue area; landscaping; and fencing.	*Study site:* Non-randomised, 1 intervention park selected for renovation by local authorities. Control park matched for similar characteristics	System for Observing Play and Recreation in Communities (SOPARC) for 9 days for each of the study conditions at three time points 6 months apart	Descriptive analysis	*Park usage:* Number of children and teen park users at baseline (14 and 57, respectively) increased post-intervention at 6 months (89 and 122) and 12 months (65 and 359) (*p*-values not provided)
**Road traffic safety**								
Dimaggio et al.			Safe Routes To School multi-component programme	Sidewalk improvements, bicycle lanes and safe crossings, improvements to signage, and traffic calming	*Schools*: Non-randomised selection, 30 of 124 potentially eligible sites which had been selected for Safe Routes To School programme on basis of high pedestrian injury rates compared with 1,347 schools without Safe Routes To School programme	Motor vehicle crash data and Safety and Safe Routes To School from the New York City DOT Office of Research, Implementation, and Safety in pre-intervention period (2001 to 2008) to post-intervention period (2009 to 2010)	Rates per 10,000 population for school-aged pedestrian injuries, rate differences and proportion changes	*Annual rate of school-aged pedestrian injury during school-travel hours*: Decreased 44% (95% CI: 17–65) from 8.0 injuries per 10,000 population in the pre-intervention period to 4.4 injuries per 10,000 population in the post-intervention period in census tracts with Safe Route to School interventions
The rate remained virtually unchanged in census tracts without Safe Routes To Schools Programme interventions (0%, 95% CI: –0.08 to 0.08)
Grundy et al.			Introduction of 20 mph traffic speed zones	Introduction of 20 mph traffic speed zones	*Study site*: Non-randomised, population-based study	Data from Police STATS19 data pre- and post-implementation (1986–2006) and Geographical Information System	Conditional fixed effects Poisson models	Annual average decline of 3.4% (95% CI: 3.1–3.7) for all casualties aged 0–15 years
Annual average decline of 3.9% (95% CI: 3.6–4.3) for all pedestrian casualties aged 0–15 years
Ragland et al.			Safe Routes To School multi-component programme	Install sidewalk (to avoid walking along roadway), traffic signal, dynamic advance intersection warning system, replace existing signals with pedestrian countdown signal heads, install flashing beacons, speed humps, changeable speed warning signs for individual drivers and improve drainage	93 of 313 agencies provided data (which can affect more than one school) in relation to 47 schools with Safe Routes To School programmes implemented	California Statewide Integrated Traffic Records System; Collisions occurring within 250-foot of built environment change buffer zones (programme areas) or a quarter-mile of school buffer zones (control areas) 48 months pre- and post-construction period	Random-intercept Poisson and random-intercept negative binomial regression models	*Collisions involving pedestrians/bicyclists aged 5 to 18 years*: Non significant reduction in collisions within 250 feet of the built environment change (incident rate ratio: 0.47, 95 CI: 0.20–1.12, *p*=0.09) between pre- and post-construction periods
**Multi-component, community-based initiatives**								
Dunton et al.		Move to Smart Growth communities		Greater building density, less auto-dominated form, greater non-residential land uses, fewer barriers to connectivity, more parks and playgrounds, more traffic safety and aesthetic features, and fewer physical incivilities such as graffiti and litter	*Study site:* A community developed to follow Smart Growth principles. Selection not described	Objectively measured MVPA with accelerometer data at baseline and 6 months	Generalised Estimating Equations regression model	There was no strong evidence for increase in MVPA in the Smart Growth group (from 32.75 min/day at time 1 to 42.78 min/day at time 2) than the control group (from 34.23 min/day at time 1 to 38.40 min/day at time 2 (*p*=0.51)
Economos et al. (2007)		Shape Up Somerville multicomponent intervention		Traffic calming tactics, to/from school environment, advocacy to paint crosswalks, install pedestrian crossing signs, open and renovate parks and bike racks, and extend a walking path in conjunction with a subway expansion project	*Study sites*: Non-randomised; 1 intervention community and 2 socio-demographically matched control communities	Objectively measured BMI at baseline, 1 and 2 year follow-up (Economos et al., 2007, 2013)	Multiple linear regression, accounting for covariates and clustering by community	*1-year follow-up time point:* Average change in BMI *z*-score in the intervention community was −0.10 compared to controls (95% CI: −0.12 to −0.09, *p*<0.001) (Economos et al., 2007)
Economos et al. (2013)	
*2-year follow-up time point*: Adjusted difference in BMI *z*-score change was −0.06 compared to controls (95%CI: −0.08 to −0.04, *p*<0.05) (Economos et al., 2013)
*Study schools*: All 30 eligible schools participated (10 intervention arm, 20 control arm);
Folta et al.	
Parent-reported reduced sugar-sweetened beverage consumption (−2.0 ounces per day; 95% CI: −3.8 to −0.2) (Folta et al., 2013)
Chomitz et al.	
Parent/caregiver report using a 68-item Family Survey Form (fruit and vegetable and sugar-sweetened beverage consumption; number of organised sports and physical activities per year; walking to and from school and screen time at baseline, 1- and 2-year follow-up (Folta et al., 2013)
Parent-reported increased participation in organised sports and physical activities (0.20 sports or activities per year; 95% CI: 0.06–0.33) (Folta et al., 2013)
*Parents:* All parents of eligible students who completed the questionnaire
Parents reported reduced screen time by their children (−0.24 h per day; 95% CI: −0.42 to −0.06).
*Students*: All consenting eligible students at schools (Economos et al., 2007, 2013)
*4-year follow-up time point*: High-school aged students were more likely to meet physical activity recommendations at follow-up after adjusting for demographic, health, and behavioural variables (OR: 1.61, 95% CI 1.34–1.92) (Chomitz et al. 2012)
Student self-report moderate or vigorous physical activity guidelines measure using Youth Risk Behaviour Surveys at baseline and 4 years follow-up (Chomitz et al., 2012)
Chi-square and logistic regression modelling
Hendricks et al.		3 prong community initiative		New sidewalks, cross-walks, crossing pedestrian signs and median islands were installed	*Study schools:* Not described	Student self-report of mode of travel for one week at baseline, 1-, 2-, and 3-year follow-up	Descriptive analysis	Four schools with at least two years data showed increases in proportion of children who walk to school. School A: 4.7% in 2004 to 12% in 2007; School B: 3% in 2005 to 9% in 2007; School C: 15% in 2005 to 30% in 2007; School D: 6% in 2006 to 9% in 2007
*Students:* Not described
Maddock et al.		The Healthy Hawaii Initiative		Planning and renovating walking paths; safe routes to schools	*Students:* Representative samples of students in grades 6–8 and 9–12 with active parental consent at all schools	Student self-report moderate or vigorous physical activity guidelines measure using Youth Risk Behaviour Surveys at baseline and 1 year, and 4 years follow-up	Not reported	*4 year follow-up time point:* The proportion of students who were overweight or at-risk for overweight increased by 2.0%; proportion of students who consumed five or more servings of fruit and vegetables a day decreased by 4.8%; no changes to proportion of students engaging in regular, moderate physical activity (*p*-values not reported)
**Active travel**								
Boarnet et al. (2005a)	Safe Routes To School multi-component programme			*Sidewalk improvements*: Construction of new sidewalks, filling gaps in the sidewalk network, construction of a walking path, and the installation of curbs and curb cuts	*Schools*: Convenience sample of 10 of 25 (40%) eligible schools that agreed to participate	Retrospective parent survey of changes to their child’s active travel 1–18 months following implementation of the programme (Boarnet et al., 2005a)	Two-sample *t*-tests	*Active travel:* Parent-reported data showed that children walked or bicycled less (18.0%, 155/862) after construction of project than children that walked or bicycled more (10.6%, 91/862) (Boarnet et al., 2005a)
Counts of walking by on-site observations pre- and post- construction of project (Boarnet et al., 2005b)
		
*Parents*: 1244 of 3222 (39%) eligible parents participated
*Built environment intervention*: Parent-reported data showed greater increase in walking level for sidewalk improvements (17.0%, 39/230, *p*<0.01) and traffic control projects, primarily traffic signals (15.9%, 21/132, *p*<0.01) if intervention was along child’s usual route to school (Boarnet et al. 2005a)
*Sidewalk improvement projects*: 3 of 5 sidewalk improvement projects (sidewalk gap closures) saw increases in walking observations (Boarnet et al., 2005b)
*Traffic signal improvement projects*: 2 traffic signal improvement projects in school sites resulted in increased walking observations (Boarnet et al., 2005b)
No observation of success for crosswalk and crosswalk signal or bicycle path improvement projects (Boarnet et al., 2005b)

*Crossing improvements*: Adding crosswalks, installing in-pavement crosswalk lighting, and installing a pedestrian activated, “count-down” street-crossing signal that warns pedestrians of the amount of time remaining to cross
*Traffic control*: Installation of a traffic signal

1Boarnet et al. (2005b)
Buliung et al.	Multicomponent School Travel Plan			Installation or repainting of crosswalk lines, removal of physical barriers preventing access to sidewalks and walkways (shrubbery and snow), installation of 4-way stops and streetlights, repair of damaged walk ways, and increases to school zone signage	*Schools*: 12 elementary schools were purposively selected based on school's willingness to participate;	Student self-report of school transport mode at baseline and approximately 1-year follow-up	Descriptive analysis	*Active travel:* Children-reported rates of active transportation increased from 43.8% to 45.9% at 1-year follow-up point (*p*-value not reported)
13.3% of families reported less driving at 1-year follow-up time point
*Students:* All students present on day of data collection
Family survey of child transportation mode at 1-year follow-up time point
Deehr et al.	Safe Routes to School as part of Active Seattle programme			Enforcement of speed limits	Not reported	Not reported	Not reported	A 24% increase in the number of students who walked to school (n or p-value not reported)
Fitzhugh et al.	Retrofitting of urban greenway/trail			Construction of a 8-foot-wide and 2.9-mile-long asphalt greenway to provide pedestrian-friendly links among residences, businesses, schools, and other public spaces	*Communities:* One intervention neighbourhood and two control neighbourhoods	Direct observation for 2 days between 7am to 9am and 2.30 pm to 4.00 pm at baseline and 2-year follow-up	Fisher exact tests	No difference in counts of active travel in experimental schools at baseline (8.5) and at 2-year follow-up time point (9.0) (*p*-values not provided)
*Schools:* 6 schools were selected. 3 intervention (2 elementary and 1 high school) and 3 control (2 elementary and 1 middle school). Criteria not described.
Garrard et al.	Ride2School multicomponent programme			Provision of facilities (bike storage) and road traffic improvements (signs and crossings)	*Study sites*: 13 (100%) schools participating in programme took part	Student self-report survey on day of data collection and previous 4 days and parent survey at baseline and approximately 8 months follow-up	Multivariate analysis	*Active travel:* Parent-reported data showed a small increase in the proportion of active trips to and from school from baseline to follow-up approximately 8 months later (47.9–49.6%) (*p*-values not reported)
*Students*: All grades 4–6 students attending participating schools were invited to participate
Student-reported data indicated a small decrease approximately 8 months later (51.1–48.7%)(*p*-values not reported)
Hinckson et al. (2011a)	School Travel Plan multicomponent programme			Crossings, sidewalks, speed bumps, signage	*Schools*: Non-randomised, all students at participating elementary schools that implemented the School Travel Plan programme	*Mode of travel*: Student survey of mode of travel on day of data collection at baseline and 1-year, 2-year, 3-year and 4-year follow-up (Hinckson et al., 2011a, 2011b)	Repeated measures logistic regression analysis	*Active travel:* Student-reported data showed by the second year of programme implementation, there was an increase in active travel by 5.9% (±6.8%)
Hinckson et al. (2011b)
Student-reported data showed by the third year of School Travel Plan implementation, there was an increase of active travel (40.5–42.2%) (OR: 2.65, 95% CI:1.75–4.02) (Hinckson et al., 2011a)
Students attending higher socioeconomic schools background showed greater improvements (38.9% to 39.1%) compared to those from mid (OR: 0.66, 95% CI: 0.82–1.01) and lower (OR: 0.47, 95% CI: 0.32–0.68) socioeconomic schools (Hinckson et al., 2011b)
Mammen et al. (2014a)	School Travel Plan multicomponent programme			Signage relating school zones, cross walks, stop signs, side walk implementations, altered drop off/pick-up zones& traffic/speed calming	*Study sites*: Non-randomised, schools that participated in the programme and provided data (53 of 106, 50%)	*Mode of travel*: Student self-report of mode of travel to school for five consecutive days at baseline and 1 year follow-up (Mammen et al., 2014a)	Backward linear regression model and binomial regression models	*Active travel:* Student-reported data showed there was no increase in active school travel at 1-year follow-up time point (baseline 27%, follow-up 31%, *n* and *p*-values not provided) (Mammen et al. 2014a)
Mammen et al. (2014b)
*Students:* Children attending school on day of data collection (Mammen et al., 2014a)	the morning (16.7% , *n*=1118) and afternoon (17.1%, *n*=1211) periods at the 1 year follow-up time point (Mammen et al., 2014b)
*Parents*: All parents with children attending schools implementing programme were invited to participate at 1 year follow-up (Mammen et al., 2014a, 2014b)	Infrastructure improvements and safety education were perceived by families as the most effective strategies implemented (Mammen et al., 2014b)
Retrospective, post-intervention parent survey at 1-year follow-up (Mammen et al., 2014b)
Schools that collected baseline data in the Fall and follow-up data in Winter saw a decrease of active travel by up to 5% (*p*<0.05) (Mammen et al., 2014a)
		Parent-reported data showed less driving in
Children’s age, household distance, and middle class neighbourhoods schools were predictors of change (Mammen et al., 2014b)
McDonald et al.	Safe Routes To School multicomponent programme			*Infrastructure improvements*: Side-walk construction, crosswalks, traffic signal improvements and placement of speed feedback trailers near schools	*Study sites*: Non-randomised, 9 schools that received funding for Safe Routes To School programme and 5 control schools (selection not described)	*School trip travel mode*: National Center for Safe Routes To School's student travel tally sheet on three consecutive days at baseline, 1, 2, 3, and 4 years follow-up	Panel fractional logit	*Active travel:* Student self-reported data showed increased walking and biking for school travel (number or *p*-values not reported)
Programmes implementing education and 2 Safe Routes to School Interventions was associated with 20% increase in walking (*p*≤0.05) but no effect on biking (*p*-value not reported)
No changes were observed in programmes which included education and crosswalks/sidewalks interventions (*p*-values not reported)
*School characteristics*: Safe Travel to School intervention combinations
Morrison et al.	Traffic calming scheme			Five sets of speed cushions (raised platforms on the road to slow car drivers), two zebra crossings with adjacent railings, and creation of parking bays	*Study site:* Non randomised	Pedestrian counts of children aged below 16 pre- and post-intervention at three locations on the site	Descriptive analysis	Site 1: Pedestrian count increased by 18% (95% CI: 15.4–20.6); Site 2: Pedestrian count increased by 44.1% (95% CI: 40.8–47.4); Site 3: Pedestrian count increased by 40.0% (95% CI: 36.9–43.1)
Moudon et al.	Safe Routes To School multicomponent programme			Sidewalk, crosswalks, signage, bicycle rack, traffic calming/control, American’s with Disabilities Act improvement, shared use path, bicycle lane and pedestrian overpass	*Study sites*: Non-randomised, all participating schools that provided data in five states	Changes in rates of Active Travel to School	Rates of change, paired sample *t*-tests and bivariate analysis	Across all projects and schools with pre- and post-project travel data in the four states, walking increased by 45% (from 9.8% to 14.2%, *p*<0.001), bicycling increased by 24% (from 2.5% to 3.0%, *p*=0.01), and all active travel modes increased by 37% (from 12.9% to 17.6%, *p*<0.001)
No significant relationships between Safe Routes To Schools project characteristics and change in rate of active school travel
Parker et al.	Addition of bike lane in an urban area			Addition of bike lane in one street	*Study site*: Non-randomised; selection of site which was having a bike lane added	Cyclist counts for 10 consecutive days at baseline and 1-year follow-up	Negative binomial regression and binary logistic regression models	Observed number of youth cycling each day increased from 2.2 (SD 3.1) at baseline to 5.2 (SD 7.4) at 1 year follow-up
Wen et al.	Central Sydney Walk to School Research programme			Working with councils to improve safety and walkability of schools and their vicinities. (No details of infrastructure reported.)	*Study sites*: Randomised, recruitment until sample size reached (*n*=24), random assignment of school to intervention or control group	*Mode of travel*: Parent reported mode of travel at baseline and at 1-year follow-up	Binary logistic regression modelling	*Active travel:* Parent-reported data showed that 29% of students in the intervention group increased their walking, compared with 19% in the control group (net increase of 9.8%, *p*=0.05) at 1-year-follow up point
There was no evidence for differences in active travel by student-reported data
*Students*: All students aged 10–12 years present in participating schools on data collection days	Student self-report by classroom survey for five consecutive days at baseline and 1-year follow-up
Student-reported data showed distance from home to school and non-car use at baseline were predictors of non-car use at 1-year-follow-up time point (both *p*<0.001)

**Table 3:** Assessment of bias in primary studies.

**Study Author**	**Assessment of bias**							
	Bias due to confounding	Bias in selection of participants	Bias in measurement of interventions	Bias due to departures from intended interventions	Bias due to missing data	Bias in measurement outcomes	Bias in selection of the reported result	**Overall bias judgment**
**Parks and playgrounds**								
Bohn-Goldbaum et al.	Serious	Serious	Low	Low	Serious	Serious	Moderate	Serious
Cohen et al. (2009)	Serious	Serious	Low	Low	Serious	Serious	Moderate	Serious
Cohen et al.	Serious	Serious	Low	Low	Serious	Serious	Moderate	Serious
Davidson et al.	Moderate	Serious	Low	Low	Low	Low	Low	Serious
Quigg et al.	Moderate	Serious	Low	Low	Low	Low	Low	Serious
Roemmich et al.	Serious	Not indicated	Low	Low	Serious	Serious	Moderate	Serious
Tester et al.	Serious	Serious	Low	Low	Serious	Serious	Moderate	Serious
Veitch et al.	Serious	Serious	Low	Low	Serious	Serious	Moderate	Serious
**Road traffic safety**								
Dimaggio, et al.	Moderate	Moderate	Low	Moderate	Low	Low	Low	Moderate
Grundy, et al.	Moderate	Low	Low	Low	Low	Low	Low	Moderate
Ragland, et al.	Moderate	Moderate	Low	Moderate	Low	Low	Low	Moderate
**Multi-component, community-based initiatives**								
Dunton, et al.	Moderate	Low	Low	Low	Low	Low	Low	Moderate
Economos, et al.	Moderate	Moderate	Low	Low	Moderate	Low	Low	Moderate
Economos, et al.	Moderate	Moderate	Low	Low	Moderate	Low	Low	Moderate
Folta, et al.	Moderate	Moderate	Low	Low	Moderate	Serious	Moderate	Serious
Chomitz, et al.	Moderate	Moderate	Low	Low	Moderate	Serious	Low	Serious
Hendricks, et al.	Serious	Serious	Moderate	Not indicated	Serious	Serious	Moderate	Serious
Maddock, et al.	Serious	Serious	Moderate	Low	Not indicated	Serious	Serious	Serious
**Active travel**								
Boarnet et al. (2005a, 2005b)	Serious	Serious	Low	Not indicated	Serious	Serious	Moderate	Serious
Buliung et al.	Serious	Serious	Low	Not indicated	Serious	Serious	Moderate	Serious
Deehr et al.	Serious	Serious	Moderate	Not indicated	Serious	Serious	Serious	Serious
Fitzhugh et al.	Serious	Serious	Moderate	Not indicated	Moderate	Serious	Moderate	Serious
Garrard J, et al.	Serious	Serious	Low	Not indicated	Serious	Serious	Moderate	Serious
Hinckson et al. (2011a, 2011b)	Moderate	Serious	Low	Not indicated	Serious	Serious	Moderate	Serious
Mammen et al. (2014a, 2014b)	Moderate	Serious	Low	Not indicated	Serious	Serious	Moderate	Serious
McDonald N, et al.	Moderate	Serious	Low	Low	Serious	Serious	Serious	Serious
Morrison et al.	Serious	Serious	Low	Low	Serious	Serious	Moderate	Serious
Moudon et al.	Serious	Serious	Low	Not indicated	Serious	Not indicated	Moderate	Serious
Parker et al.	Serious	Serious	Moderate	Low	Serious	Serious	Moderate	Serious
Wen et al.	Moderate	Low	Low	Low	Serious	Serious	Low	Serious


**Table 4:** Summary of results and risk of bias for included studies.

**Study**	**Key reported results**		**Overall risk of bias**
**Park or playground improvements**			
Bohn-Goldbaum et al.	No difference in children’s park usage; no difference in children’s MVPA; decline in girls’ MVPA in intervention park	x	Serious
Cohen et al. (2009)	Number of children and adolescent park users declined	x	Serious
Cohen et al. (2012)	Number of children and adolescent park users declined	x	Serious
Davidson et al.	Rates of injuries in children aged 5–16 years decreased	✓	Serious
Quigg et al.	No differences in mean total daily PA; children with higher BMI more likely to reduce PA	x	Serious
Roemmich et al.	Odds of children standing or being in MVPA rather than sitting not associated with intervention	x	Serious
Tester et al.	Number of children and teen park users increased	✓	Serious
Veitch et al.	Number of children and teen park users increased	✓	Serious
**Road safety measures**			
Dimaggio et al.	School-aged pedestrian injury during school-travel hours decreased	✓	Moderate
Grundy et al.	Decline for all casualties aged 0–15 years; decline for all pedestrian casualties aged 0 to 15 years	✓	Moderate
Ragland et al.	Non-significant reduction in collisions involving pedestrians/bicyclists aged 5 to 18 years	✓	Moderate
**Multi-component community-based initiatives**			
Dunton et al.	No strong evidence for increase in MVPA	x	Moderate
Economos et al. (2007, 2013)	Reduction in BMI *z*-score	✓	Moderate
Folta et al.	Parent-reported children’s: reduced sugar-sweetened beverage consumption; increased PA; reduced screen time	✓	Serious
Chomitz et al.	High-school students more likely to meet PA recommendations	✓	Serious
Hendricks et al.	Increases in proportion of children who walk to school	✓	Serious
Maddock et al.	Proportion of students overweight/at-risk of overweight increased; proportion of students consuming 5 or more servings of fruit and vegetables a day decreased; no changes to proportion of students engaging in regular moderate PA	x	Serious
**Active travel**			
Boarnet et al. (2005a, 2005b)	Parent-reported children walking/ bicycling less; increased walking observed in 3 of 5 sidewalk improvement projects, and 2 traffic signal improvement projects	x✓	Serious
Buliung et al.	Child-reported active travel increased; families reported less driving	✓	Serious
Deehr et al.	Increase in students who walked to school	✓	Serious
Fitzhugh et al.	No difference in counts of active travel	x	Serious
Garrard et al.	Parent-reported increase in student active travel; student-reported decrease in active travel	✓	Serious
x
Hinckson and Badland (2011), Hinckson et al. (2011)	Student-reported increase in active travel	✓	Serious
Mammen et al. (2014a, 2014b)	Student-reported no increase in active travel; parent-reported less driving	x	Serious
✓
McDonald et al.	Overall student self-reported data showed increased walking and biking for school travel	✓	Serious
Morrison et al.	Pedestrian counts increased	✓	Serious
Moudon et al.	Walking, cycling and all active travel modes increased	✓	Serious
Parker et al.	Increase in observed youth cycling each day	✓	Serious
Wen et al.	Parent-reported increase in students walking; no evidence for differences in active travel by student-reported data	✓	Serious
x
