# Supplementary material for: Healthy urban environments for children and young people: A systematic review of intervention studies
Source: Health Place. 2015 Nov;36:97–117. doi: 10.1016/j.healthplace.2015.09.004 (PMC4676191; doi:10.1016/j.healthplace.2015.09.004)
Supplement: Supplementary file 1 — Supplementary material [file mmc1.docx]

*Supplementary file 1. Search strategy applied to Medline database*

1. OBESITY/
2. WEIGHT GAIN/
3. WEIGHT LOSS/
4. obes$.tw.
5. (weight gain or weight loss).tw.
6. (overweight or over weight or overeat$ or over eat$).tw.
7. weight change$.tw.
8. ((bmi or body mass index) adj2 (gain or loss or change)).tw.
9. 1 or 2 or 3 or 4 or 5 or 6 or 7 or 8
10. DIET/
11. DIETARY FATS/
12. ((calori* or fat? or fatty or fizz* or soft* or carbonated* or sweetened or salt* or sugar* or fruit* or veg? or vegetable* or fibre* or fiber* or 5-a-day or five a day or go for 2&5) adj2 (intake or food* or diet* or consum* or meal* or eat* or nutrition or drink* or snack*)).tw.
13. ((poor or over* or unhealthy or health*) adj3 (nutrition or diet* or eat* or meal* or food* or snack* or drink*)).tw.
14. 10 or 11 or 12 or 13
15. EXERCISE/
16. PHYSICAL ACTIVITY/
17. (physical activity or physical exert* or exercise or aerobic activit* or sport* or active lifestyle* or outdoor activit* or gym* or mvpa sport$ or walk$ or bicycle$).tw.
18. (exercise$ adj3 aerobic$).tw.
19. ((cycle or cycling) adj5 (school* or commut* or travel* or equipment or facilit* or rack*1 or store*1 or storing or park* or friendly or infrastructure)).ti,ab.
20. ((lifestyle or life-style) adj5 activ$).tw.
21. ((lifestyle or life-style) adj5 physical$).tw.
22. ((promot* or uptak* or encourag* or increas* or start* or adher* or sustain* or maintain*) adj5 physical activ*).tw.
23. ((decreas* or reduc* or discourag*) adj5 (sedentary or deskbound or physical inactiv*)).tw.
24. (active adj (travel*4 or transport* or commut*)).tw.
25. ((moderate or vigorous*) adj activ*).tw.
26. (physical adj5 (fit* or train* or activ* or endur* or exerc*)).tw.
27. (exercis* adj5 (fit* or train* or activ* or endur* or aerobic)).tw.
28. 15 or 16 or 17 or 18 or 19 or 20 or 21 or 22 or 23 or 24 or 25 or 26 or 27
29. MENTAL DISORDERS/
30. ADJUSTMENT DISORDERS/
31. ANXIETY DISORDERS/
32. MOOD DISORDERS/
33. NEUROTIC DISORDERS/
34. (anxi* or depress* or melancholi* or neuros* or neurotic or psychoneuro* or stress* or distress* or emotion* or well-being or well being or affective symptom* or Good Childhood Index).tw.
35. 29 or 30 or 31 or 32 or 33 or 34
36. CHILD/
37. ADOLESCENT/
38. CHILD, PRESCHOOL/
39. INFANT/
40. (child$ or pediatr$ or paediatr$ or adolescen* or teen* or youth* or hooligan or young adult* or early adult* or juvenile* or minor? or emerging adult* or girl$ or boy$ or youth or youths or apprentice* or FE college* or young m$n or young wom$n or young male* or young female* or young people or young person or young adult$ or under 18* or schoolchild$ or school child$ or sixth-form* or secondary education or tertiary education or higher education or further education or preschool* or primary education or infan* or kid or nurser* or playschool* or kindergarten* or prekindergarten*).tw.
41. (teacher* or parent* or guardian* or grandparent* or mother* or father* or mum$1 or dad$1 or maternal or paternal or nurse? or childminder or child care provider or playworker or family or families or carer*).tw.
42. 36 or 37 or 38 or 39 or 40 or 41
43. URBAN HEALTH/
44. CITY PLANNING/
45. URBAN RENEWAL/
46. ENVIRONMENT DESIGN/
47. PUBLIC FACILITIES/
48. (physical environment or urban environment or built environment or school environment).tw.
49. (population density or street connectivity or neighbo$rhood or residence characteristics).tw.
50. (urban design or urban population or bicycle paths or pavement or pedestrian crossing$ or safe routes or sidewalk or traffic calming or traffic lights or pedestrian*).tw.
51. (open space$ or playing field$ or green space$ or greenspace$ or greenness or greenery or park activit$ or parkland or park improvement$ or public park$ or playground$ or public space or open space or recreation or recess or outdoor).tw.
52. (crime or street violence or mugged or mugging or robbery or vandalism or assaults or fear of crime).tw. (((food environment or nutrition environment or food retail) and access) or neighborhood destinations or food store availability or grocery store).tw.
53. (((food environment or nutrition environment or food retail) and access) or neighborhood destinations or food store availability or grocery store).tw.
54. 43 or 44 or 45 or 46 or 47 or 48 or 49 or 50 or 51 or 52 or 53
55. INTERVENTION STUDIES/
56. EVALUATION STUDIES/
57. PROGRAM EVALUATION/
58. (comparative study or quasi-experiment$ or trial or intervention group or control group or mixed methods or longitudinal or natural experiment).tw.
59. (time adj series).tw.
60. ((pre adj test) or pretest or ((posttest or post) adj test)).tw.
61. ((evaluat$ or intervention or interventional) adj8 (control or controlled or study or program$ or comparison or (before adj2 after adj2 study) or comparative)).tw.
62. ((intervention or interventional) adj8 (effect$ or evaluat$ or outcome$)).tw.
63. ((process or program$) adj3 (effect$ or evaluat$)).tw.
64. (follow adj up adj assessment).tw.
65. (controlled adj before).tw.
66. (before adj2 after adj2 study).tw.
67. 55 or 56 or 57 or 58 or 59 or 60 or 61 or 62 or 63 or 64 or 65 or 66
68. 9 or 14 or 28 or 35
69. 42 and 54 and 67 and 68
